# Retraction Note: Azoramide, a novel regulator, favors adipogenesis against osteogenesis through inhibiting the GLP-1 receptor-PKA-β-catenin pathway

**DOI:** 10.1186/s13287-023-03513-2

**Published:** 2023-09-29

**Authors:** Banjun Ruan, Zheng Zhu, Zhao Yan, Wei Yang, Dongsheng Zhai, Li Wang, Zichen Ye, Huanyu Lu, An Xiang, Jingwei Liang, Yinghao Jiang, Chengming Xu, Zhenyu Wang, Ming Wei, Xiaoying Lei, Xiaorui Cao, Zifan Lu

**Affiliations:** 1 State Key Laboratory of Cancer Biology, Department of Pharmacogenomics, Fourth Military Medical University, Xi’an, 710032 People’s Republic of China; 2 Institute of Urinary Surgery, Xijing Hospital, Fourth Military Medical, University, Xi’an, 710032 People’s Republic of China; 3 Institute of Orthopedic Surgery, Xijing Hospital, Fourth Military Medical University, Xi’an, 710032 People’s Republic of China; 4 Infection and Immunity Program, Department of Microbiology, Biomedicine Discovery Institute, Monash University, Clayton, VIC 3800 Australia; 5 Department of Occupational and Environmental Health and the Ministry of Education Key Lab of Hazard Assessment and Control in Special Operational Environment, School of Public Health, Fourth Military Medical University, Xi’an, 710032 People’s Republic of China; 6 School of Pharmacy, China Medical University, Liaoning, 110122 China; 7 Department of Pharmacology, Xi’an Medical University, Xi’an, 710021 People’s Republic of China

## Retraction Note: Stem Cell Research & Therapy (2018) 9:57 10.1186/s13287-018-0771-y

The Editors-in-Chief have retracted this article at the authors' request. After publication, the authors found errors in the following figure panels:

- Fig. 2a: The representative ALP staining images of 15 and 30 μM are both chosen from the 30 μM group
- Figs. 4d, 5h and 5i: The same β-actin blot is used.
- Fig. 4e and f: The same β-actin blot is used
- Fig. 5a: The representative ALP staining images of Azo+si-glp-1r-1 and Azo+si-glp-1r-2 are the same

The authors provided the raw data for validation. However, further checks by the Publisher identified some differences between the raw data and the figures, as well as high similarity between the original gel images for Fig. 5b GLP-1 and 5i Fabp4. The Editors-in-Chief and the authors therefore no longer have confidence in the presented data.

All authors agree to this retraction.

